# Widening prospective and current medical students' participation in research

**DOI:** 10.1111/medu.14779

**Published:** 2022-02-24

**Authors:** Lara Gracie, Danial Rostami‐Hochaghan, Basel Taweel, Nasir Mirza

## 1 WHAT PROBLEM WAS ADDRESSED?

*First problem*. Less privileged sixth form (high school) students get fewer opportunities to undertake extracurricular academic activities that will enhance their competitiveness for entry into medical schools.

*Second problem*. Restricted access to healthcare settings because of the COVID‐19 pandemic meant that sixth form students were unable to obtain medicine‐related work experience.

*Third problem*. Learning research skills is an important part of medical training. It is challenging for students to find research projects to participate in, and many students wishing to participate in research projects are unable to do so. Research opportunities for students became even more limited during the COVID‐19 pandemic, as laboratory and departmental occupancy levels were restricted.

*Fourth problem*. Genetics tests and treatments are becoming an increasingly common part of medical practice, but medical students' knowledge of genetics is often poor. Medical students' knowledge of neurology, including epilepsy, is also often poor.

*Fifth problem*. There are no up‐to‐date epilepsy gene databases; creating one will help accelerate mechanistic/therapeutic discovery in epilepsy.

## 2 WHAT WAS TRIED?

A Medline search for studies reporting genes associated with epilepsy revealed ~10 000 abstracts, which were manually screened to identify relevant abstracts and extract data from them. Students were invited to perform the manual screening and data extraction. Headmasters of three schools—a fee‐paying school, a ‘typical’ state school and a state school with a higher‐than‐average number of students from an economically deprived background—were contacted to publicise the project amongst their sixth form students. The project was also publicised amongst medical students by word‐of‐mouth. Over 60 students from multiple schools and universities in different cities took part. Detailed written instructions explaining genetic and epilepsy concepts were created and then optimised based upon feedback from a focus group of students. An introductory YouTube video was created by a medical student. Sixth form students were invited to live virtual tutorials. Manual curation was performed using an online systematic review tool (www.sysrev.com). An administrative team (comprised of three medical students, supervised by a senior clinical lecturer) performed live audits of students' initial responses and provided individualised feedback. The extracted data were collated by a medical student into an online database (www.liverpool.ac.uk/D3RE/SAGAS; to be described in a separate publication, which will have all the students listed as co‐authors).

## 3 WHAT LESSONS WERE LEARNED?

Aspiring/current medical students are able to learn about advanced genetic and medical terminologies and successfully apply them in data‐mining research. Such large‐scale online‐only projects enable many students from multiple institutions in different cities to participate in research, even during a pandemic. Live audit of participants' early results and providing individualised feedback is feasible and aids student education and research efficiency. About 90% of students felt that their understanding of genetics, epilepsy and research improved. Sixth form students felt that their applications to medical school would be strengthened. Participation and completion rates were lowest for sixth form students from the state school with a higher‐than‐average number of pupils from an economically deprived background. In future, there should be more strenuous outreach/support to less privileged students.

